# Association of childhood trauma, and resilience, with quality of life in patients seeking treatment at a psychiatry outpatient: A cross-sectional study from Nepal

**DOI:** 10.1371/journal.pone.0275637

**Published:** 2022-10-04

**Authors:** Saraswati Dhungana, Rishav Koirala, Saroj Prasad Ojha, Suraj Bahadur Thapa

**Affiliations:** 1 Department of Psychiatry and Mental Health, Institute of Medicine, Tribhuvan University, Kathmandu, Nepal; 2 Division of Mental Health and Addiction, Institute of Clinical Medicine, University of Oslo, Oslo, Norway; 3 Brain and Neuroscience Center, Kathmandu, Nepal; 4 Division of Mental Health and Addiction, Oslo University Hospital, Oslo, Norway; The Royal Melbourne Hospital, AUSTRALIA

## Abstract

Quality of life is defined by the World Health Organization as "Individuals’ perception of their position in life in the context of the culture and value systems in which they live and in relation to their goals, expectations, standards and concerns". It is a comprehensive measure of health outcome after trauma. Childhood maltreatment is a determinant of poor mental health and quality of life. Resilience, however, is supposed to be protective. Our aim is to examine childhood trauma and resilience in patients visiting psychiatry outpatient and investigate their relations with quality of life. A descriptive cross-sectional study was conducted with a hundred patients with trauma and visiting psychiatry outpatient. Standardized tools were applied to explore childhood trauma, resilience, quality of life and clinical diagnoses and trauma categorization. Sociodemographic and relevant clinical information were obtained with a structured proforma. Bivariate followed by multivariate logistic regressions were conducted to explore the relation between childhood trauma, resilience, and quality of life. Poor quality of life was reported in almost one third of the patients. Upper socioeconomic status, emotional neglect during childhood, current depression and low resilience were the determinants of poor quality of life in bivariate analysis. Final models revealed that emotional neglect during childhood and low resilience had independent associations with poor quality of life. Efforts should be made to minimize childhood maltreatment in general; and explore strategies to build resilience suited to the cultural context to improve quality of life.

## Introduction

Childhood maltreatment has been defined “as any act or series of acts of commission or omission by a parent or other caregiver that results in harm, potential for harm, or threat of harm to a child” [1]. This has been studied extensively in the last few decades [2, 3]. Studies have revealed that childhood maltreatment is not an uncommon occurrence with most children exposed to at least one form of maltreatment [4–6]. Reported prevalence rates of childhood maltreatment in high income countries vary from one country to another, ranging from 5–6% for both sexes in Norway to 27% girls and 30% in boys for physical abuse in the United States. Similarly, the rates of sexual abuse reported was 10–14% in girls and 3–4% in boys in the Norwegian general population, while it was higher for boys up to 6% and for girls as high as 14% in a meta-analysis conducted in Europe in 2011, with even higher rates in the US, 25% in girls and 16% in boys [5, 7–10]. Studies on prevalence and forms of maltreatment in the low- and middle-income countries context are scant with rates even higher [11]. A study from India among 12–18 years adolescents with history of child workforce reported rates above 80% for any form of maltreatment, while physical abuse was the most common form reported in almost two-thirds of the participants [12]. Studies from Nepal report similar estimates with some variations on the predominant form of trauma [13–15].

Childhood maltreatment has long-term adverse consequences on health through a number of pathways, extending from biological networks to psychological vulnerability [16]. Most studies point towards a consistent, and negative correlation between childhood trauma and a number of adverse physical and mental health conditions, including quality of life [7, 13, 17–19], adding on to the societal burden and economic costs [7, 20, 21]. Cumulative traumatic events in context of childhood trauma have been linked to further adversities later in life [22] and impaired quality of life [18, 23].

On the other hand, resilience has been found to have a protective effect on later mental health outcomes [24]. In face of the highly prevalent nature of trauma in general population globally, resilience is key in maintaining an optimal health outcome [25]. Resilience is, therefore, a key component in trauma research [25] although there are discussions on the complex and multisystemic nature of resilience [24, 26]. Individuals who are resilient are said to have better mental health profiles [24, 27], including better quality of life.

Quality of life (QOL) is defined by the World Health Organization as "Individuals’ perception of their position in life in the context of the culture and value systems in which they live and in relation to their goals, expectations, standards and concerns". It therefore, is a multidimensional construct of health that goes beyond clinical diagnoses. Studies suggest that there is relationship between resilience and quality of life [27] but the nature of the relation between the two has not been studied extensively, especially in context of trauma and childhood trauma.

Nepal is a lower- middle income country in South Asia, with 17.4% of the population still under poverty line as of 2021. Studies suggest that children who have been exposed to poverty and adverse social circumstances are more at risk of experiencing childhood traumatic events [3, 28, 29]. Furthermore, physical acts of discipline are common practices in children and young people embedded within the culture with similar rates of emotional and other traumatic events [13, 30] in Nepali population. To our knowledge, scant literature is available from Nepal [13–15, 30] on childhood maltreatment prevalence and correlates but no studies exploring relation with QOL.

The current study thereby attempts to fulfill this gap by exploring the prevalent types of childhood trauma individuals report when they come to seek treatment at a psychiatric outpatient of a university hospital in Kathmandu, Nepal. We also investigated the relationship between most kinds of childhood trauma, resilience, and quality of life in these individuals. We hypothesized that QOL is poor in those with positive childhood trauma history and has positive relation with resilience.

## Methods

### Participants

This study had a cross sectional descriptive design and was a part of a broader study “Study of health outcomes after trauma” (SHOT) [31, 32]. The participants in this study were adults 18–60 years presenting to psychiatry outpatient of Tribhuvan University Teaching hospital, a tertiary hospital in Kathmandu, Nepal. The eligibility criteria were at least one trauma exposure before one month prior to the outpatient visit, operationalized as defined in the ICD-10 PTSD section K [33]. Those with dyslexia, cerebral infection, severe head injury, serious medical or neurological illness, organic mental disorders, and psychotic disorders were excluded from the study. We used purposive sampling technique for the study participants. Sample size was calculated based on the Cochran’s formula of sample size calculation for cross- sectional studies as follows:

$$\text{n}={\left(\text{z}1-\alpha /2\right)}^{2}{}^{*}\left(\text{p}\right)\left(\text{q}\right)/{\left(\text{d}\right)}^{2},\text{n}={\left(\text{z}1-\alpha /2\right)}^{2}{}^{*}\left(\text{p}\right)\left(\text{q}\right)/{\left(\text{d}\right)}^{2}.$$

Here, p = 50% from previous study [34], (z_1−_ α _/2_)^2^ = 1.96 for 95% confidence interval, and d = 10%, precision of estimate. With this, the sample size calculated, n = 96.04 ~ 96. Considering 10% dropouts, we calculated the total required sample size as 96+10 = 106. We, however, were able to recruit only 100 patients in our study. However, for a few participants, observations on some variables were missing. We had missing information on childhood trauma for two patients and on resilience and QOL measure for one patient so we have used the data from 98 of the participants for childhood trauma measure and 99 for QOL and resilience measure.

### Procedure

Patients fulfilling the inclusion criteria for the study and who consented to participate were provided detailed information regarding the project in verbal and in written form. They were also informed about their voluntariness in willing to withdraw from the study at any time during data collection without the need for justification.

Written informed consent was then obtained from all participants deemed eligible for the study. Informants accompanying the participants provided consent for those not able to read and / or write. Ethical approval for this study was obtained from the Nepal Health Research Council (NHRC) (reference number 801) and Institutional Review Committee (IRC) at the Institute of Medicine (reference number 480 (611)6^2^/ 075/076) in Nepal and Research Ethical Committee (REK) in Norway (reference number 2015/2081). Details about the methodology are published elsewhere [32, 35].

A predesigned proforma was used to gather sociodemographic and other relevant information on trauma. We categorized gender as binary, age into two groups as less or equal to 24 and more than 24 years, marital status into two as single and married, religion as Hindu and others, residence as rural and urban, education as illiterate and literate and socioeconomic status into two as lower and upper. Trauma variables of interest were trauma numbers into two as single and repetitive and time since trauma elapsed into three as trauma less than 1 year, 1–10 years and more than 10 years.

### Study measures

#### WHO Quality of Life-Brief version (WHOQOL-BREF)

The WHOQOL-BREF is a shorter 26- item version of the original WHOQOL 100-item scale. It is a comprehensive measure of QOL incorporating multiple domains, with good psychometric properties. The total scores are computed by summing up the scores on each domain as specified in the manual [36]. The Nepali translation by Giri et al. [37] was used in this study, where we categorized the total QOL score into two categories as good (total QOL score more than 45) and poor (total QOL score less or equal to 45) [38].

#### Child Trauma Questionnaire Short Form (CTQ-SF)

The Childhood Trauma Questionnaire-Short Form (CTQ-SF) [39] is the gold standard in regard to assessing childhood traumas and encompasses five aspects, namely emotional abuse, physical abuse, emotional neglect, physical neglect, and sexual abuse. It is brief, convenient with good psychometric properties across clinical samples as a retrospective measure of childhood maltreatment. For our purpose, we used the Nepali translated and adapted version by Kohrt et al. [40, 41]. This version excluded the statements referring to sexual abuse for cultural adaptation purposes. In Nepali culture, questions regarding sexual abuse are considered inappropriate and offensive even to this date, making the participants uncomfortable. The cut-off score used for both physical neglect and physical abuse was nine, whereas the cut-off for emotional abuse was twelve and emotional neglect was fourteen as reported in the original.

#### Wagnild and Young Resilience scale (RS)

The Wagnild and Young Resilience scale (RS) is a shorter eight item version of the original 25- item scale [42]. The original scale comprises two domains, namely personal competence with seventeen items and acceptance of self and life domain with eight items, respectively. For our study, we used the Nepali translated and adapted version [41]. This is a Likert type scale, where higher mean scores point towards better resilience.

#### WHO World Mental Health Composite International Diagnostic Interview (WMH-CIDI) version 2.1

WHO-CIDI 2.1 is a standardized diagnostic interview guide developed by WHO, which corresponds to psychiatric diagnoses in ICD-10 and DSM-IV. The psychometric properties are reported to be good in studies [33]. We used the Nepali translation with standard guidelines [43]. We used sections D, E and K for diagnoses of generalized anxiety disorder, major depressive disorder, and post-traumatic stress disorder, respectively. We also used the list of ten traumatic events listed in section K to categorize trauma.

### Statistics

Stata 17 was used for all statistical purposes (Stata Corp LLC, College Station, TX, USA). Normal distribution of continuous variables was assessed using histogram and boxplots. Then after, means, medians, standard deviations and inter quartile ranges were applied for continuous variables and frequencies with percent for categorical variables for descriptive statistics. For inferential statistics, chi-square tests were applied for bivariate association between categorical variables followed by multivariate logistic regression in the final models. Variables found significant in the bivariate analyses, along with other relevant variables from literature such as age, gender, and current psychiatric diagnoses, were first checked for confounding and those with variation inflation factor less than 2 were entered in the final models to test our hypotheses. Hosmer and Lemeshow test were done to check for model fit. P value .05 was considered significant for all statistical purposes.

## Results

There were 99 participants in this study. Almost one-third of the participants had a poor quality of life. Upper socioeconomic status and emotional neglect type of childhood trauma along with low resilience and current major depression had significant associations with poor QOL.

### Sociodemographic profile of participants and QOL

Table 1 shows the sociodemographic correlates of participants and QOL.

**Table 1:** Sociodemographic variables and quality of life.

Variables (*n = 99*)	Quality of life		α^2^	CI (LB, UB)
	Poor(33)	Better(66)		
Gender				
Male 48	16	32	-0.0	.43, 2.33
Female 51	17	34		
Age (years)				
Less or equal to 24	5	14	0.52	.22, 2.03
More than 24	28	52		
Marital status				
Single	5	15	0.78	.20, 1.27
Married	28	51		
Religion				
Hindu	28	53	0.30	.44, 2.44
Others	5	13		
Residence				
Rural	19	30	1.30	.70, 0.38
Urban	14	36		
Education				
Illiterate	7	10	0.55	0.52, 4.40
Literate	26	56		
SES				
Lower	14	44	5.32*	.16, 0.87
Upper	19	22		

α^2^ = chi- square statistic, CI = Confidence interval, LB = Lower bound, UB = Upper bound, SES = socioeconomic status

*p<0.05

### Childhood trauma types, time since trauma, other trauma and QOL

Table 2 depicts the association of childhood trauma types, time since trauma, number of traumas and QOL.

**Table 2:** Child trauma, trauma variables and quality of life.

Variables (*n = 98*)	QOL		α^2^	CI (LB, UB)
	Poor	Better		
Trauma number				
Single	15	26	0.33	0.55, 2.98
Repetitive	18	40		
Time since trauma in years				
Less than 1	5	9	0.51	
1–10	23	50		.36, 4.00
More than 10	5	7		.16, 3.8
CTQ				
CTQ Physical abuse				
No	23	46	0.05	0.44, 2.82
Yes	9	20		
CTQ Emotional abuse				
No	24	56	1.34	0.19, 1.52
Yes	8	10		
CTQ Physical neglect				
No	13	28	0.03	0.39, 2.19
Yes	19	38		
CTQ Emotional neglect				
No	22	58	4.97*	0.11, 0.87
Yes	10	8		

n = number of participants, α^2^ = chi-square statistic, CI = Confidence interval LB = lower bound, UB = upper bound, CTQ = child trauma questionnaire, QOL = quality of life,

*p<0.05

### Current psychiatric disorders and QOL

Table 3 shows the association of psychiatric disorders, and QOL.

**Table 3:** Psychiatric disorders and QOL.

Variables (*n = 98*)	QOL		α^2^	CI (LB, UB)
	Poor	Better		
PTSD				
No	27	56	0.15	0.26, 2.44
Yes	6	10		
Depression				
No	11	56	26.44***	0.03,0.24
Yes	22	10		
GAD				
No	17	44	2.11	0.23, 1.25
Yes	16	22		
PTSD+depression				
No	29	60	0.22	0.19, 2.77
Yes	4	6		
PTSD+GAD				
No	31	61	0.08	0.23, 6.92
Yes	2	5		

n = number of participants, α^2^ = chi-square statistic, CI = Confidence interval, LB = Lower bound, UB = Upper bound, PTSD = Post traumatic stress disorder, GAD = Generalized anxiety disorder, PTSD = post-traumatic stress disorder, QOL = quality of life,

***p<0.001

### Factors independently associated with QOL

Table 4 shows all the factors associated with QOL. Bivariate analyses were first done followed by multivariate logistic regressions to check if childhood traumatic events and resilience had statistically significant associations in predicting QOL. Variables that were shown to be statistically significant in the bivariate models were entered into the final models along with other relevant variables such as age, gender, and other current psychiatric diagnoses from published literature. After adjustment in the final models, only childhood trauma type emotional neglect, resilience scores and current depression were found to have significant associations with better QOL.

**Table 4:** Factors associated with better QOL.

Variables (*n = 98*)	Bivariate analysis (Unadjusted model)	Multivariate analysis (Adjusted model)
	OR (CI)	OR (CI)
Age (≤24 year)	0.66 (0.22, 2.03)	.17 (0.02, 1.25)
Gender (Male)	1 (0.43, 2.31)	0.96 (0.25, 3.72)
SES (Lower)	0.37 (0.16, 0.87)*	0.61 (0.16, 2.34)
CTQ Emotional neglect (No)	0.30 (0.11, -0.87)*	0.07 (0.01, 0.43)**
Current depression (No)	0.09 (0.03, 0.24)***	0.05 (0.01, 0.26)***
Current PTSD (No)	0.80 (0.26, 2.44)	2.62 (0.46, 14.80)
Current GAD (No)	0.53 (0.23, 1.25)	0.87 (0.21, 3.55)
Mean resilience score	(2.13, 6.44)***	4.52 (1.97, 10.40)***

n = number of participants, OR = odds ratio, CI = Confidence interval, CTQ = child trauma questionnaire, PTSD = post-traumatic stress disorder, GAD = generalized anxiety disorder, SES = socio-economic status,

*p<0.05

**p<0.01

***p<0.001

## Discussion

This study explores QOL in context of childhood trauma and resilience taken into consideration in the context of Nepal. The findings from this study extend support to the existing literature suggesting poor quality of life in patients with childhood trauma. Almost one-third of patients who sought treatment in psychiatry outpatient department reported poor quality of life. Though literature on exact prevalence on QOL estimates is scant, studies report impaired QOL in survivors of major trauma [44]. Many factors contribute to poor QOL in such studies.

Our study concluded that QOL was better in those with lower socioeconomic status in bivariate analyses. This is in striking contrast to most studies reporting a positive correlation between the two [45, 46]. SES is a robust measure of health comprising three most essential elements, namely education, employment, and family income [47]. It is the single most important social determinant of overall health and quality of life. However, the association is complex and is a function of a number of biological, psychological, social, and cultural factors [48]. Exploring other confounders might have given a more accurate picture. The possibility of having more depression/ emotional neglect/ childhood trauma or other relevant factors such as social support, in those with higher SES is also a valid concern prone to give biased estimates in terms of association with QOL. Also, the nature of the measure both in terms of SES and the QOL and the context of their usage need to be considered before making generalization. The rest of the sociodemographic variables did not show statistical significance.

It is interesting to note that those with history of emotional neglect form of trauma in childhood had independent association with poor quality of life in our study, with other traumas having no effect. This was a surprising result since several studies examining the relationship between various childhood trauma types and adult QOL, including systemic reviews [18, 49] have reported otherwise. Childhood trauma is related to poor QOL regardless of the type of trauma. This was also one of our hypotheses on starting to write the paper, but during analysis, we found that only emotional neglect was associated with QOL, unlike in most studies. Psychological maltreatment, a broader term encompasses both abuse and neglect and has been shown to be associated with an equal and even greater number of behavioral problems and disorders [50]. Studies have consistently demonstrated a clear relationship between most kinds of childhood trauma [16, 18, 51], ranging from physical abuse, physical neglect, emotional neglect, emotional abuse, and sexual abuse [2, 16]. Neglect of any kind is as harmful as physical and sexual abuse though has received less attention [8]. QOL assessment during childhood and adolescence also demonstrated clear negative association with any kind of childhood maltreatment [52, 53]. It is important to note that these effects extend beyond adulthood [54]. This could be for the fact that there is heterogeneity in terms of the measure of childhood trauma and childhood maltreatment, as much as the study design and study population. One of the most influential papers in childhood maltreatment and its trajectory later in life studied multiple forms of maltreatment, where many family problems such as mental illness in family, mothers exposed to violence were also given same scores as other forms such as neglect or abuse [4]. Biased estimates in terms of maltreatment reporting are also possible depending on whether it is self-reported or parental report [3]. Ours is a study conducted in those seeking treatment at psychiatry facility and there was also history of another trauma considered besides the childhood trauma variable. As much as the presence of childhood trauma, we also know that the perception of trauma, the nature of trauma on whether it is a recurrent event, social circumstances of trauma and more importantly, social support following trauma are more important considerations in the trajectory of childhood maltreatment [3, 22].

Another finding that needs discussion here is the association of resilience with QOL. High score on resilience measure was independently associated with better QOL, even after adjusting for all other relevant factors. This is in line with most studies examining resilience and QOL in context of trauma population with lower resilience meaning lower QOL [55, 56]. One of these studies was among earthquake survivors of earthquake in China, which reported association between resilience and QOL, however, the relation was partly mediated by social support [55]. A systemic review conducted among conflict- driven adults with forced migration reported that resilience was generally related to better mental health in displaced populations with a caution that the evidence was limited [57]. Furthermore, another study from Toronto in homeless and mentally ill adults concluded that resilience and quality of life were positively correlated even after adjusting for major demographic and clinical parameters [27].

A study conducted in China among patients with bladder cancer reported that resilience was a key factor along with social support and hope, that predicted QOL, though the nature of trauma was different compared to ours [58]. Studies exploring resilience, however, need to be examined critically since resilience has differing perspectives and can be studied from a multisystem approach, especially in relation to trauma and quality of life [26].

Current depression was the only clinical diagnosis independently associated with QOL outcome. This is in line with several studies after trauma exposure exploring QOL [59]. However, the finding of current generalized anxiety disorder and PTSD not related to QOL is in contrast with results from other studies [60–62]. Also, generalized anxiety disorder is usually not diagnosed separately or investigated when it comes to trauma related disorders, instead it is categorized broadly under stress and trauma related disorders. In the ICD-10 system, the diagnosis is hierarchical, which allows to make a diagnosis of depression leaving PTSD and GAD, if depression criteria are fulfilled. Again, the fact that this was a hospital-based study with small sample size could be another explanation limiting the comparison with existing studies. There is, however, robust evidence that diagnoses of anxiety disorders have significant impairment in QOL and needs to be taken into consideration [63].

This study, however, is not without limitations and therefore, the results should be interpreted with caution. Child trauma questionnaire we used though was adapted in Nepali context, the whole chunk of sexual trauma related questions was removed saying they were not appropriate to ask in the cultural context. The possibility of recall and response bias cannot be excluded as well since this was self-reported measure as well as retrospective measure. This might have confounded the estimates. Although most of the tools we used in this study such as WHOQOL-BREF, CTQ- SF and RS were translated and adapted versions already used in Nepal, they have their own culture specific dimensions. This should also be taken into consideration in interpreting the results. This is a hospital- based study of those seeking help and with history of another trauma with cross-sectional design, limiting the casual explanation and the directionality of association. Some of the estimates could have been significant due to the small sample size. Despite these, this study attempts to explore the relation between resilience and QOL in context of trauma and childhood trauma and is possibly the first in our setting. We firmly believe that this will serve as a reference in guiding similar studies on a larger scale.

## Conclusion

Almost one third of trauma exposed patients seeking treatment at psychiatry outpatient had poor quality of life. Emotional neglect during childhood and current depression diagnosed currently were independent predictors of poor QOL, while resilience was protective. Therefore, resilience building treatment packages should be the goal. We recommend longitudinal studies with better methodology and validated tools in our context to get more clarity on this critical area.

## Supporting information

S1 Dataset (DTA)

## References

[1] Leeb RT. Child maltreatment surveillance: Uniform definitions for public health and recommended data elements. Centers for Disease Control and Prevention, National Center for Injury Centers for Disease Control and Prevention; 2008.

[2] Downing NR, Akinlotan M, Thornhill CW. The impact of childhood sexual abuse and adverse childhood experiences on adult health related quality of life. Child Abuse & Neglect 120 (2021) 105181. doi: 10.1016/j.chiabu.2021.105181 34247038

[3] Beal SJ, Wingrove T, Mara CA, et al. Childhood Adversity and Associated Psychosocial Function in Adolescents with Complex Trauma. Child & Youth Care Forum. 2019;48(3):305–322. doi: 10.1007/s10566-018-9479-5 31467469 PMC6715307

[4] Felitti VJ, Anda RF, Nordenberg D, et al. Relationship of childhood abuse and household dysfunction to many of the leading causes of death in adults. The Adverse Childhood Experiences (ACE) Study. Am J Prev Med. 1998;14(4):245–58. doi: 10.1016/s0749-3797(98)00017-8 9635069

[5] Merrick MT, Ford DC, Ports KA, et al. Prevalence of adverse childhood experiences from the 2011–2014 behavioral risk factor surveillance system in 23 states. JAMA Pediatrics. 2018;172(11):1038–1044. doi: 10.1001/jamapediatrics.2018.2537 30242348 PMC6248156

[6] Stoltenborgh M, Bakermans-Kranenburg MJ, Alink LRA, et al. The Prevalence of Child Maltreatment across the Globe: Review of a Series of Meta-Analyses. Child Abuse Review. 2015;24(1):37–50.

[7] Greger HK, Myhre AK, Lydersen S, et al. Child maltreatment and quality of life: a study of adolescents in residential care. Health and Quality of Life Outcomes. 2016;14(1). doi: 10.1186/s12955-016-0479-6 27161357 PMC4862063

[8] Gilbert R, Widom CS, Browne K, et al. Burden and consequences of child maltreatment in high-income countries. The Lancet. 2009;373(9657):68–81. doi: 10.1016/S0140-6736(08)61706-7 19056114

[9] Stoltenborgh M, Van Ijzendoorn MH, Euser EM, et al. A global perspective on child sexual abuse: Meta-analysis of prevalence around the world. Child Maltreatment. 2011;16(2):79–101. doi: 10.1177/1077559511403920 21511741

[10] Gervin DW, Holland KM, Ottley PG, et al. Centers for Disease Control and Prevention Investments in Adverse Childhood Experience Prevention Efforts. American Journal of Preventive Medicine. 2022;62(6):S1–S5. doi: 10.1016/j.amepre.2021.11.014 35597578 PMC11219185

[11] Cuartas J, McCoy DC, Rey-Guerra C, et al. Early childhood exposure to non-violent discipline and physical and psychological aggression in low- and middle-income countries: National, regional, and global prevalence estimates. Child Abuse & Neglect. 2019;92:93–105. 30939376 10.1016/j.chiabu.2019.03.021

[12] Pandey R, Gupta S, Upadhyay A, et al. Childhood maltreatment and its mental health consequences among Indian adolescents with a history of child work. Australian & New Zealand Journal of Psychiatry. 2020;54(5):496–508. doi: 10.1177/0004867420909524 32156147 PMC7227131

[13] Dhakal S, Niraula S, Sharma NP, et al. History of abuse and neglect and their associations with mental health in rescued child labourers in Nepal. Australian & New Zealand Journal of Psychiatry. 2019;53(12):1199–1207. doi: 10.1177/0004867419853882 31185738

[14] Neupane D, Bhandari PM, Thapa K, et al. Self-reported child abuse in the home: a cross-sectional survey of prevalence, perpetrator characteristics and correlates among public secondary school students in Kathmandu, Nepal. BMJ Open. 2018;8(6):e018922. doi: 10.1136/bmjopen-2017-018922 29921678 PMC6009519

[15] Atteraya MS, Ebrahim NB, Gnawali S. Determinants of child maltreatment in Nepal: Results from the 2014 Nepal multiple indicator cluster survey (the 2014 NMICS). Child Abuse & Neglect. 2018;76:400–407. 29232623 10.1016/j.chiabu.2017.11.018

[16] Corso PS, Edwards VJ, Fang X, et al. Health-Related Quality of Life Among Adults Who Experienced Maltreatment During Childhood. American Journal of Public Health. 2008;98(6):1094–1100. doi: 10.2105/AJPH.2007.119826 18445797 PMC2377283

[17] Bosch J, Mackintosh M-A, Wells SY, et al. PTSD treatment response and quality of life in women with childhood trauma histories. Psychological Trauma: Theory, Research, Practice, and Policy. 2020;12(1):55–63. doi: 10.1037/tra0000468 31081656

[18] Weber S, Jud A, Landolt MA. Quality of life in maltreated children and adult survivors of child maltreatment: a systematic review. Quality of Life Research. 2016;25(2):237–255. doi: 10.1007/s11136-015-1085-5 26245708

[19] Norman RE, Byambaa M, De R, et al. The Long-Term Health Consequences of Child Physical Abuse, Emotional Abuse, and Neglect: A Systematic Review and Meta-Analysis. PLoS Medicine. 2012;9(11):e1001349. doi: 10.1371/journal.pmed.1001349 23209385 PMC3507962

[20] Fang X, Brown DS, Florence CS, et al. The economic burden of child maltreatment in the United States and implications for prevention. Child Abuse Negl. 2012;36(2):156–65. doi: 10.1016/j.chiabu.2011.10.006 22300910 PMC3776454

[21] Brown DS, Fang X, Florence CS. Medical Costs Attributable to Child Maltreatment. American Journal of Preventive Medicine. 2011;41(6):627–635. 22099241 10.1016/j.amepre.2011.08.013

[22] Hughes K, Bellis MA, Hardcastle KA, et al. The effect of multiple adverse childhood experiences on health: a systematic review and meta-analysis. The Lancet Public Health. 2017;2(8):e356–e366. doi: 10.1016/S2468-2667(17)30118-4 29253477

[23] Wu NS, Schairer LC, Dellor E, et al. Childhood trauma and health outcomes in adults with comorbid substance abuse and mental health disorders. Addictive Behaviors. 2010;35(1):68–71. doi: 10.1016/j.addbeh.2009.09.003 19775820 PMC3666315

[24] Horn SR, Charney DS, Feder A. Understanding resilience: New approaches for preventing and treating PTSD. Experimental Neurology. 2016;284:119–132. doi: 10.1016/j.expneurol.2016.07.002 27417856

[25] Peterson KA, Zhou L, Watzlaf VJM. A Comprehensive Review of Quality of Life Surveys for Trauma-Affected Communities. Perspect Health Inf Manag. 2019;16(Winter):1e. 30766456 PMC6341418

[26] Ungar M, Theron L. Resilience and mental health: how multisystemic processes contribute to positive outcomes. The Lancet Psychiatry. 2020;7(5):441–448. doi: 10.1016/S2215-0366(19)30434-1 31806473

[27] Mejia-Lancheros C, Woodhall-Melnik J, Wang R, et al. Associations of resilience with quality of life levels in adults experiencing homelessness and mental illness: a longitudinal study. Health and Quality of Life Outcomes. 2021;19(1): p. 74. doi: 10.1186/s12955-021-01713-z 33663538 PMC7971123

[28] Walsh D, McCartney G, Smith M, et al. Relationship between childhood socioeconomic position and adverse childhood experiences (ACEs): a systematic review. Journal of Epidemiology and Community Health. 2019;73(12):1087–1093. doi: 10.1136/jech-2019-212738 31563897 PMC6872440

[29] Benjet C. Childhood adversities of populations living in low-income countries: prevalence, characteristics, and mental health consequences. Curr Opin Psychiatry. 2010;23(4):356–62. doi: 10.1097/YCO.0b013e32833ad79b 20520546

[30] Kandel P, Kunwar R, Karki S, et al. Child maltreatment in Nepal: prevalence and associated factors. Public Health. 2017;151:106–113. doi: 10.1016/j.puhe.2017.06.020 28763786

[31] Søegaard EGI, Kan Z, Koirala R, et al. Variations in psychiatric morbidity between traumatized Norwegian, refugees and other immigrant patients in Oslo. Nordic Journal of Psychiatry. 2020;74(6):390–399. doi: 10.1080/08039488.2020.1714724 31961250

[32] Koirala R, Søegaard EGI, Ojha SP, et al. Trauma related psychiatric disorders and their correlates in a clinical sample: A cross-sectional study in trauma affected patients visiting a psychiatric clinic in Nepal. PLoS one. 2020;15(6):e0234203. doi: 10.1371/journal.pone.0234203 32541999 PMC7295578

[33] Andrews G, Peters L. The psychometric properties of the Composite International Diagnostic Interview. Social Psychiatry and Psychiatric Epidemiology. 1998;33:80–88. doi: 10.1007/s001270050026 9503991

[34] Thapa SB and Hauff E. Psychological distress among displaced persons during an armed conflict in Nepal. Social Psychiatry and Psychiatric Epidemiology, 2005. 40(8): 672–679. doi: 10.1007/s00127-005-0943-9 16021344

[35] Dhungana S, Koirala R, Ojha SP, et al. Quality of life and its association with psychiatric disorders in outpatients with trauma history in a tertiary hospital in Kathmandu, Nepal: a cross-sectional study. BMC Psychiatry. 2021;21(1). 10.1186/s12888-021-03104-6 PMC7885479 33593325

[36] Program on Mental Health: WHOQOL user manual: 2012 revision: World Health Organization 1998.

[37] Giri S, Neupane M, Pant S, et al. Quality of life among people living with acquired immune deficiency syndrome receiving anti-retroviral therapy: a study from Nepal. HIV/AIDS (Auckland, NZ). 2013;5:277. doi: 10.2147/HIV.S50726 24101885 PMC3790835

[38] Attafuah PYA, Everink I, Abuosi AA, et al. Quality of life of older adults and associated factors in Ghanaian urban slums: a cross-sectional study. BMJ Open. 2022;12(2):e057264. doi: 10.1136/bmjopen-2021-057264 35149573 PMC8845194

[39] Bernstein DP, Stein JA, Newcomb MD, et al. Development and validation of a brief screening version of the Childhood Trauma Questionnaire. Child Abuse Negl. 2003;27(2):169–90. doi: 10.1016/s0145-2134(02)00541-0 12615092

[40] Kohrt BA, Worthman CM, Adhikari RP, et al. Psychological resilience and the gene regulatory impact of posttraumatic stress in Nepali child soldiers. Proceedings of the National Academy of Sciences 2016;113(29):8156–8161. doi: 10.1073/pnas.1601301113 27402736 PMC4961140

[41] Kohrt BA, Worthman CM, Ressler KJ, et al. Cross-cultural gene− environment interactions in depression, post-traumatic stress disorder, and the cortisol awakening response: FKBP5 polymorphisms and childhood trauma in South Asia. International Review of Psychiatry. 2015;27(3):180–196. doi: 10.3109/09540261.2015.1020052 26100613 PMC4623577

[42] Wagnild GM, Young HM. Development and psychometric evaluation of the resilience scale. Journal of Nursing Measurement. 1993;1(2):165–17847. 7850498

[43] Van Ommeren M, Sharma B, Thapa S, et al. Preparing instruments for transcultural research: use of the translation monitoring form with Nepali-speaking Bhutanese refugees. Transcultural Psychiatry. 1999;36(3):285–301.

[44] Danielsson FB, Schultz Larsen M, Nørgaard B, et al. Quality of life and level of post-traumatic stress disorder among trauma patients: A comparative study between a regional and a university hospital. Scandinavian Journal of Trauma, Resuscitation and Emergency Medicine. 2018;26(1). doi: 10.1186/s13049-018-0507-0 29859111 PMC5984827

[45] Kim J-H, Park E-C. Impact of socioeconomic status and subjective social class on overall and health-related quality of life. BMC Public Health. 2015;15(1). 10.1186/s12889-015-2014-9 PMC4536592 26275823

[46] Thumboo J, Fong K-Y, Machin D, et al. Quality of life in an urban Asian population: the impact of ethnicity and socio-economic status. Social Science & Medicine. 2003 2003/04/01/;56(8):1761–1772. 12639592 10.1016/s0277-9536(02)00171-5

[47] Kagamimori S, Gaina A, Nasermoaddeli A. Socioeconomic status and health in the Japanese population. Social Science & Medicine. 2009;68(12):2152–2160. 10.1016/j.socscimed.2009.03.030 19375838

[48] Adler NE, Boyce T, Chesney MA, et al. Socioeconomic status and health: The challenge of the gradient. American Psychologist. 1994;49(1):15–24. 8122813 10.1037//0003-066x.49.1.15

[49] Abajobir AA, Kisely S, Williams G, et al. Does substantiated childhood maltreatment lead to poor quality of life in young adulthood? Evidence from an Australian birth cohort study. Quality of Life Research. 2017;26(7):1697–1702. doi: 10.1007/s11136-017-1517-5 28236264

[50] Spinazzola J, Hodgdon H, Liang L-J, et al. Unseen wounds: The contribution of psychological maltreatment to child and adolescent mental health and risk outcomes. Psychological Trauma: Theory, Research, Practice, and Policy. 2014;6(Suppl 1):S18–S28.

[51] Piontek K, Wiesmann U, Apfelbacher C, et al. The association of childhood maltreatment, somatization and health-related quality of life in adult age: Results from a population-based cohort study. Child Abuse & Neglect. 2021;120:105226. doi: 10.1016/j.chiabu.2021.105226 34352685

[52] Vink RM, van Dommelen P, van der Pal SM, et al. Self-reported adverse childhood experiences and quality of life among children in the two last grades of Dutch elementary education. Child Abuse & Neglect. 2019;95:104051. doi: 10.1016/j.chiabu.2019.104051 31344586

[53] Ustuner Top F, Cam HH. Childhood maltreatment among university students in Turkey: prevalence, demographic factors, and health-related quality of life consequences. Psychol Health Med. 2021;26(5):543–554. doi: 10.1080/13548506.2020.1768274 32432899

[54] Draper B, Pfaff JJ, Pirkis J, et al. Long-Term Effects of Childhood Abuse on the Quality of Life and Health of Older People: Results from the Depression and Early Prevention of Suicide in General Practice Project. Journal of the American Geriatrics Society. 2008;56(2):262–271. doi: 10.1111/j.1532-5415.2007.01537.x 18031482

[55] Xu J, Ou L. Resilience and quality of life among Wenchuan earthquake survivors: the mediating role of social support. Public Health. 2014;128(5):430–437. doi: 10.1016/j.puhe.2014.03.002 24792190

[56] Imai R, Hori H, Itoh M, et al. Relationships of blood proinflammatory markers with psychological resilience and quality of life in civilian women with posttraumatic stress disorder. Scientific Reports. 2019;9(1). doi: 10.1038/s41598-019-54508-0 31784695 PMC6884570

[57] Siriwardhana C., et al., A systematic review of resilience and mental health outcomes of conflict-driven adult forced migrants. Conflict and Health, 2014. 8(1): p. 13. doi: 10.1186/1752-1505-8-13 25177360 PMC4149800

[58] Li M-Y, Yang Y-L, Liu L, et al. Effects of social support, hope and resilience on quality of life among Chinese bladder cancer patients: a cross-sectional study. Health and Quality of Life Outcomes. 2016;14(1). doi: 10.1186/s12955-016-0481-z 27153944 PMC4859956

[59] Shieh V, Huang JJ, Wu TG, et al. Rate of psychiatric disorders and associations with quality of life among community members following the Kaohsiung gas explosion: an 18-month cross-sectional follow-up study. Health Qual Life Outcomes. 201917(1):1:0. doi: 10.1186/s12955-018-1076-7 30635003 PMC6330396

[60] Siqveland J, Nygaard E, Hussain A, et al. Posttraumatic growth, depression and posttraumatic stress in relation to quality of life in tsunami survivors: a longitudinal study. Health and Quality of Life Outcomes. 2015;13(1):18. doi: 10.1186/s12955-014-0202-4 25889940 PMC4326430

[61] Bras M, Milunovic V, Boban M, et al. Quality of life in Croatian Homeland war (1991–1995) veterans who suffer from post-traumatic stress disorder and chronic pain. Health Qual Life Outcomes. 2011;9(1):1–8. doi: 10.1186/1477-7525-9-56 21798076 PMC3157409

[62] Nygaard E, Heir T. World assumptions, posttraumatic stress and quality of life after a natural disaster: A longitudinal study. Health and Quality of Life Outcomes. 2012;10(1):76. doi: 10.1186/1477-7525-10-76 22742447 PMC3478202

[63] Mendlowicz MV. Quality of Life in Individuals With Anxiety Disorders. American Journal of Psychiatry. 2000;157(5):669–682. doi: 10.1176/appi.ajp.157.5.669 10784456

